# Control and Manipulation of Pathogens with an Optical Trap for Live Cell Imaging of Intercellular Interactions

**DOI:** 10.1371/journal.pone.0015215

**Published:** 2010-12-31

**Authors:** Jenny M. Tam, Carlos E. Castro, Robert J. W. Heath, Michael L. Cardenas, Ramnik J. Xavier, Matthew J. Lang, Jatin M. Vyas

**Affiliations:** 1 Division of Infectious Disease, Massachusetts General Hospital, Harvard Medical School, Boston, Massachusetts, United States of America; 2 Mechanical Engineering and Biological Engineering, Massachusetts Institute of Technology, Cambridge, Massachusetts, United States of America; 3 Center for Computational and Integrative Biology, Massachusetts General Hospital, Harvard Medical School, Boston, Massachusetts, United States of America; Singapore Immunology Network, A*STAR, Singapore

## Abstract

The application of live cell imaging allows direct visualization of the dynamic interactions between cells of the immune system. Some preliminary observations challenge long-held beliefs about immune responses to microorganisms; however, the lack of spatial and temporal control between the phagocytic cell and microbe has rendered focused observations into the initial interactions of host response to pathogens difficult. This paper outlines a method that advances live cell imaging by integrating a spinning disk confocal microscope with an optical trap, also known as an optical tweezer, in order to provide exquisite spatial and temporal control of pathogenic organisms and place them in proximity to host cells, as determined by the operator. Polymeric beads and live, pathogenic organisms (*Candida albicans* and *Aspergillus fumigatus*) were optically trapped using non-destructive forces and moved adjacent to living cells, which subsequently phagocytosed the trapped particle. High resolution, transmitted light and fluorescence-based movies established the ability to observe early events of phagocytosis in living cells. To demonstrate the broad applicability of this method to immunological studies, anti-CD3 polymeric beads were also trapped and manipulated to form synapses with T cells *in vivo*, and time-lapse imaging of synapse formation was also obtained. By providing a method to exert fine control of live pathogens with respect to immune cells, cellular interactions can be captured by fluorescence microscopy with minimal perturbation to cells and can yield powerful insight into early responses of innate and adaptive immunity.

## Introduction

Intercellular contacts, such as neurological synapses [1] and immune synapses [2], [3], are crucial in biology; however, generating and imaging physiologically relevant models of intercellular contact have proven challenging. Of particular interest is the process of phagocytosis [4], in which cells of the mammalian immune system, such as macrophages, dendritic cells, and neutrophils, physically contact a pathogen (*e.g.* bacteria and fungi) and subsequently ingest it. Successful phagocytosis not only neutralizes the threat from the pathogen, but also generates antigenic material crucial for the adaptive immune response [5]. The regulation of phagocytosis and phagosome formation has significant implications in the initiation of specific immune responses to pathogens. Thus, a detailed understanding of the molecular mechanism of this process is necessary to understand the pathogenesis of multiple types of infection.

Investigators have previously studied phagocytosis by microscopy; however, most studies suffer from one or more of the following shortcomings: (1) they frequently use polymeric beads in place of pathogenic microbes as phagocytic cargo [6], [7]; (2) they use imaging techniques such as epifluorescent microscopy, which limits the spatial resolution that can be captured in a single intercellular interaction; (3) to our knowledge, they lack the ability to closely mimic ideal physiological conditions for live cell imaging; (4) finally, and most crucially, they rely on serendipitous mixing of immune cells with pathogens to initiate phagocytosis. In this article, we describe a technique for observing immune cell contact events, which overcomes these limitations. Our method uses optical trapping to position live fungal pathogens at any arbitrary time and at any arbitrary location relative to immune cells, and allows real-time observation of the entire process using spinning disk confocal microscopy.

Current physical methods to manipulate single cells for microscopy can provide spatiotemporal control over cells [8], [9], but some techniques such as glass needle manipulation and magnetic nanowires [10], [11], permanently alter the native structure of the cells, serve to activate cells by physical contact. Microfluidic techniques [12] have also been used to position cells in solution, but most devices lack flexibility to manipulate the particle in any direction. We, therefore, use optical trapping as a non-perturbing, but fast and effective method to position cells in culture.

Optical trapping, also known as optical tweezers, is an emerging technology in the field of biological research to trap and physically manipulate cells and other micron-sized particles in three dimensions. Radiation pressure was first observed and applied [13], [14] to optical tweezer systems in 1970 [15], [16], and has been used to control biological specimens [17]. Since then, optical tweezers has matured into a technology to probe a variety of biological phenomena [18], [19], [20], [21], [22], [23].

To simultaneously trap and image microparticles, optical tweezer systems are usually built in conjunction with epifluorescent and TIRF (total internal reflection) microscope systems [24]. However, these imaging techniques are not well suited for imaging three dimensional cellular structures and processes. Recently, optical traps have started to integrate with laser scanning confocal microscopes [25], [26], [27], but the scan rate of the single laser beam is typically time consuming and too slow to image fleeting, real-time cellular processes. For example, to capture a 1024×1024 high resolution image, only two or three images could be produced per second, which may not be fast enough to capture fluorescence signals that last only a tenth of a second. In contrast, our method uses spinning disk confocal microscopy for optimal fluorescent imaging resolution and to provide fast image acquisition rates required for live cell imaging, able to capture 15–20 full frame images per second. This technique offers enough spatial and temporal resolution to capture short-lived cell-cell interactions.

We apply optical trapping to study live fungal pathogens such as *Aspergillus fumigatus* (*A. fumigatus*) and *Candida albicans* (*C. albicans*), which can cause potentially lethal, invasive infections [28], [29] in immunocompromised individuals (*e.g.* AIDS patients, chemotherapy patients, organ transplantation patients). A previous study has used optical trapping to study phagocytosis [30], [31] although no live organisms were used. In contrast to polymeric beads and other inert specimens, live pathogens recruit physiologically relevant proteins to the phagosome [32], [33] – for example, PAMP (pathogen-associated molecular pattern) receptors, CD63, CD82, and class II MHC molecules.

Although some live cell imaging has been conducted with optical tweezers [34], to our knowledge, these studies were not performed under *in vivo* conditions where physiological conditions such as temperature and CO_2_ levels were controlled. As a result, cellular interactions will behave differently. To address this limitation, we use a temperature and CO_2_ controlled and chamber where, through digital controls, the temperature and CO_2_ can be set precisely.

Historically, intercellular contact events such as phagocytosis have been imaged by mixing two cell types, and then continuously scanning the field of view for pairs of cells at the appropriate stage of interaction. This process is an inherently tedious due to the stochastic nature of these events, at least at physiologically relevant cell densities, and the kinetics of cell movement. It is particularly difficult to observe early or fleeting events in cell-cell contact by this approach, since this method requires finding cell pairs which are on the verge of contact, and observing them until they consummate their contact, or do not.

In this report, we address all the aforementioned shortcomings by describing the design and construction of an optical trapping system integrated with a spinning disk confocal microscope and describe its use to control and position live pathogens at arbitrary locations on the microscope stage over timescales of seconds. We conduct our live cell experiments in a temperature-controlled and CO_2_-controlled environment in order to mimic physiological conditions. In addition, we designed our trapping apparatus to occupy a minimal footprint in a laboratory setting – 4.5 ft^2^ – in order to apply this method in any laboratory setting. We show that by positioning these pathogens adjacent to phagocytic cells, we can initiate phagocytosis and monitor and image its outcome. To further validate the broad applicability of this method in immunology and to record intercellular interactions, we imaged and analyzed the formation of immunological synapses between cultured T lymphocytes and beads conjugated with anti-CD3. These beads were controlled and positioned by the optical trap to stimulate T cells to form protrusions and subsequent synapses. By combining optical tweezers and spinning disk confocal microscopy, live pathogens can be controlled and positioned with respect to immune cells, and visualization of intercellular interactions and responses can be conducted with minimal perturbation to the cells. Consequently, novel insight into early responses in innate and adaptive immunity can be observed.

## Results

### Experimental setup

**Figure 1:**
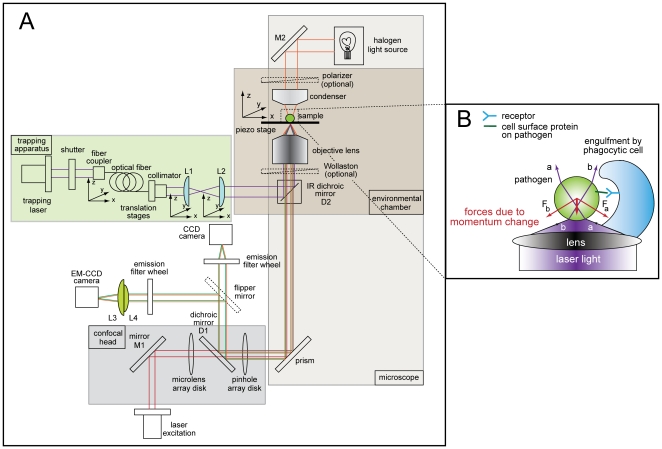
Overview and schematic of combined optical trap and spinning disk confocal microscope setup. Instrument layout (a) showing the trapping beam (purple), illumination path for brightfield imaging (orange), fluorescence excitation beam (red), fluorescence emission (green), charge-coupled device (CCD) camera, electron-multiplying charge coupled device (EM-CCD) camera, dichroic mirrors (D1 and D2), mirrors (M1 and M2), and lenses (L1, L2, L3, L4). All other components of the trap-confocal microscope system are labeled in the figure. (**b**) Detailed schematic of a trapped pathogen (green) is shown. The laser light (purple) refracts through the pathogen, creating a momentum change (red, labeled F_a_ and F_b_), pushing the pathogen up and into the focused trapping beam. As the pathogen is trapped, it can then be positioned next to a phagocytic cell (blue) where the cell surface proteins on the pathogen can engage in receptors on the phagocytic cell surface.

To exert full spatial and temporal control of pathogens and beads in an *in vivo* and *in vitro* environment, we designed a custom-built trapping apparatus integrated onto a spinning disk confocal microscope (schematic shown in Fig. 1A). A detailed discussion of the components and layout can be found in the Materials and Methods. The IR laser was chosen for its ease of setup and power output needed to create a trap sufficient to hold microorganisms or size-matched beads. The wavelength used for trapping was selected to minimize photodamage in live specimens [35] and avoid cross-talk with common emission wavelengths used in fluorescence imaging. At full strength, the laser provided 350 mW of power and after coupling the light with the various optical components in the optical trap apparatus, ∼5 mW of power at the TIRF objective, as measured with a power meter, was used to form the trap in the chambered coverglass.

By using an optical trap, a microparticle (*e.g.* cell, bead) can be manipulated non-destructively and directed towards a phagocytic cell, engaging cell surface receptors to trigger the phagocytosis process. This technique is based on radiation pressure induced by a laser beam tightly focused by a high numerical aperture lens (shown in Fig. 1B). The focused laser beam is refracted, when it enters the cell, and again when it exits. The difference in the photon momentum before and after refraction is transferred to the particle such that the resulting radiation pressure, expressed as F_a_ and F_b_, is exerted in the direction opposite to the momentum change. Force is also exerted in the downstream direction of the laser by photons that are reflected by the captured particle. The sum of the forces at each point in the particle is directed to the highest-intensity region at the focal point. Consequently, the particle is attracted to the focused beam and is trapped in the vicinity of the focal point.

In order to position an object in the chamber relative to the trapped object, the stage was moved while holding the trapped object in the stationary trapping laser. The stage was moved at slow enough velocities (as specified in each section below) so that the drag force created on the trapped particle did not exceed the maximum force generated by the optical trap.

### Trapping and positioning polymeric beads

**Figure 2:**
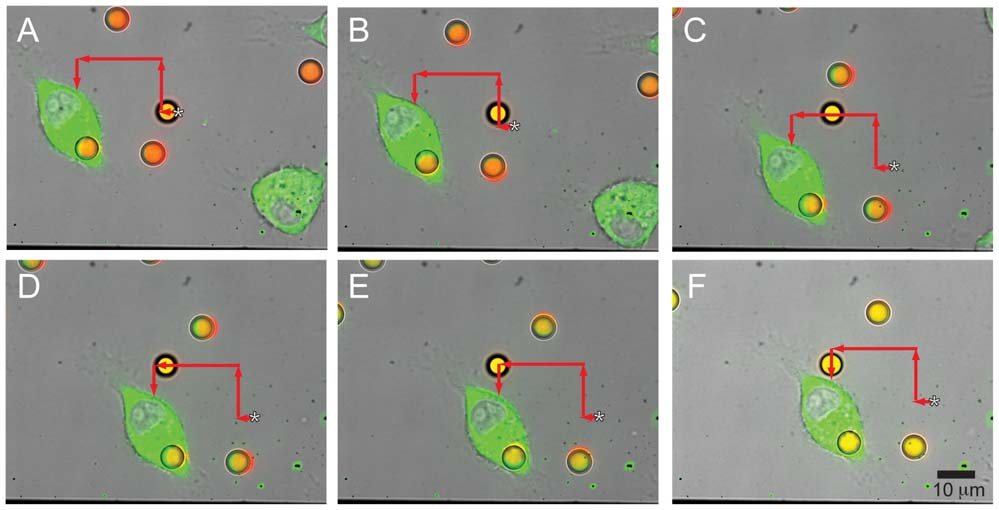
Trapping and positioning of polystyrene bead next to a fluorescent J774 cell. (a–f) Combined fluorescent and brightfield images extracted from a real-time movie of a fluorescent polystyrene bead (yellow) positioned next to a fluorescent J774 cell expressing YFP-actin (green) (see Supporting Information for Video S1). The bead is trapped in a field of other fluorescent beads (orange) and another fluorescent J774 cell (also labeled green) as the stage is moved to position the cell next to the trapped bead. The red arrows indicate the deflection of the stage to direct the bead adjacent to the J774 cell, and the starting position of the trapped bead is indicated by a star (*).

To validate our system, and to demonstrate the method of positioning particles next to cells of interest, 5 µm fluorescent polystyrene beads (labeled with “Flash Red” dye; ex: 660 nm/em: 690 nm) were optically trapped and moved along a pre-determined path as shown by the red arrows and placed adjacent to a J774 mouse macrophage expressing YFP-actin (Fig. 2 and Video S1). This system permits time-lapse imaging using simultaneous brightfield and confocal fluorescence imaging as the bead is moved next to a fluorescent cell. After the bead was trapped, the stage was moved using a joystick at an average velocity of 0.76 µm/sec in order to place the bead in the desired location. With this technique, we established a standard protocol to trap, translocate, and position a particle in a preferred location while simultaneously imaging using brightfield and confocal fluorescence microscopy.

### Mobility of *Candida albicans*

**Figure 3:**
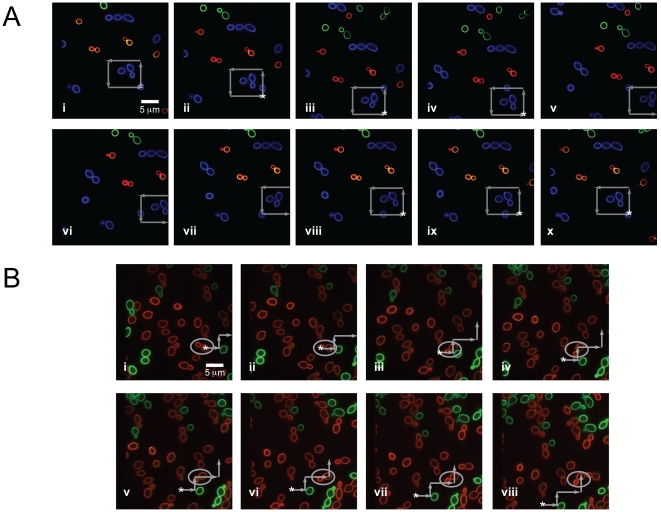
Fluorescence images of trapped and manipulated *C. albicans*. (a) A trapped fluorescently-labeled (Alexa Fluor 488 (AF488), blue) organism in a field of other fluorescently-labeled *C. albicans* (AF568, green and AF647, red). The stage is moved around the trapped, blue CA particle as indicated by the gray arrows, and follows the sequence in frames i–x. The starting position of the CA is indicated by a star (*), and briefly moves out of the field of view in frames v–vii. Full length movie of trapped *C. albicans* can be seen in Supporting Information, Video S2. (b) Trapped *C. albicans* with attached, budding daughter cell (labeled with AF647, red, and highlighted in the gray circle) in a highly dense field of AF647-labeled and AF488-labeled (green) organisms. The stage is maneuvered around the red *C. albicans*, as indicated by the gray arrows. Frames i-viii shows the sequence of movements to position *C. albicans* in a different area of the stage. The starting position of the mother-daughter pair is indicated by a star (*).

Having established a protocol for positioning polymeric beads, we next studied the trapping and repositioning of fluorescently labeled *C. albicans*. In contrast to nearly perfect spherical polymer beads, the variability in shapes of pathogenic yeast poses challenges to generating optical traps sufficient for specific displacement of the particle. Indeed, the shape of particles, not size, played a dominant role in phagocytosis by lung alveolar macrophages [36]. In addition, these organisms readily “bud” a daughter cell, which adds complexity to the trapping procedure. To test whether our designed system could specifically trap pathogenic fungi, separate populations of ∼5 µm *C. albicans* cells were labeled with each of three colors (AF488, AF568, and AF647, corresponding to green, blue, and red in the figure, respectively) to illustrate imaging with multiple fluorescence channels while simultaneously optically trapping the pathogen. A single *C. albicans* was trapped and moved in a square pattern around a cluster of other yeast, as indicated by gray arrows, demonstrating the ability to capture and manipulate the specific location of a single pathogen chosen by the operator (Fig. 3A and Video S2).

To illustrate further the flexibility of this system to trap the different shape morphologies exhibited by pathogenic organisms, the optical tweezer was also able to hold and situate a *C. albicans* particle with an attached daughter cell. In this experiment, only two labels were used: *C. albicans* labeled with AF488 (green) and *C. albicans* labeled with AF647 (red). The dimeric particle was moved in a dense field of *C. albicans* along the path, as indicated by the gray arrows (Fig. 3B and Video S3). As the trapped portion of the *C. albicans* was moved along the pre-determined path, the daughter cell also moved and was free to rotate and move around the larger trapped *C. albicans* mother particle. The particle of interest was easily maneuvered in a dense field of other pathogens without additional unintended interactions of the trap with other particles. The stage moved at an average velocity of 0.29 µm/sec in order to direct the *C. albicans* particle.

### Real time imaging of phagocytosis of *A. fumigatus: Trapping and positioning A. fumigatus*

**Figure 4:**
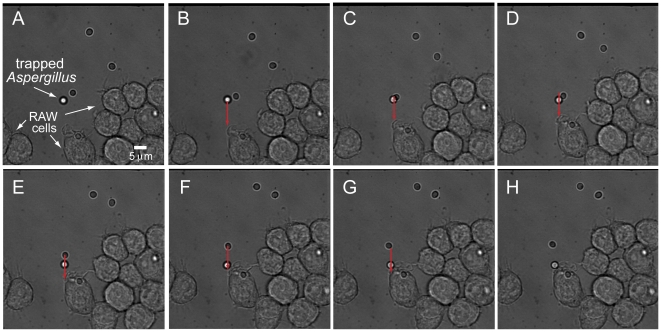
Trapping and positioning of *A. fumigatus* next to a phagocytic RAW cell. (a–g) Brightfield images of a trapped *A. fumigatus*, as indicated by the white arrow, moved and positioned along the path as indicated by the red arrow. The trapped pathogen is slightly out of focus due to the trap pushing the organism slightly above the focal plane. The trap is fine enough to select the desired particle even as another microbe moves close to the trapped organism (c). *A. fumigatus* is moved until it is placed adjacent to the desired RAW cell (h). Movie of positioned *A. fumigatus* can be seen in Video S4.

We also extended the utility of our system to trap *A. fumigatus*, a spherical yet smaller (∼3 µm diameter), fungal pathogen of significant clinical importance [37]. Despite its smaller size and propensity to adhere to the coverglass surface, we were able to position it readily next to a RAW mouse macrophage cell in order to analyze the absolute time frame of phagocytosis with this particular cell line and pathogen (Fig. 4 and Video S4). The trapped *Aspergillus* particle is pushed slightly above the coverglass surface and moved in the trajectory indicated by the red arrow. The *A. fumigatus* remains stationary as the stage is positioned such that the particle is directed adjacent to a RAW cell. Once the pathogen is placed next to the cell, the trap is turned off, and time-lapse imaging is employed to observe the subsequent cellular events. The stage moved at an average velocity of 0.49 µm/sec in order to place the pathogen next to the RAW cell.

**Figure 5:**
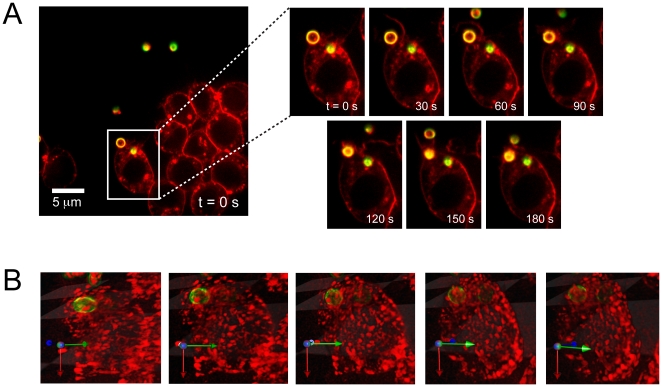
Fluorescence imaging of phagocytosis of *A. fumigatus* by RAW cell. (a) After the trapped pathogen is placed next to a RAW cell, the phagocytosis process is activated. At 30 s, the membrane of the RAW cell starts to change and form a cup around the particle. At 60 s, the cup is fully formed. From 90 s to 150 s, the *A. fumigatus* is engulfed, and by 180 s, the particle is fully internalized. Movie of complete phagocytosis of *A. fumigatus* can be seen in Video S5. (b) 3-D rendering of RAW cell with ingested *A. fumigatus*. Close-up of RAW cell to verify that the pathogen is fully phagocytosed. The cell is rotated in each successive frame, and the axis in the lower left corner of each frame shows the degree of rotation (readers are able to virtually rotate the cell in Video S6). Please note that Video S6 is a QTVR movie, which is actually an animation that is based on the user's mouse movements.

The juxtaposition of the fungal particle adjacent to the mouse macrophage initiated the phagocytic machinery and time-lapse imaging captures the entire process (Fig. 5A and Video S5). Each frame in the figure represents a 30 sec window in the phagocytic process. From the beginning of the membrane change, upon positioning the *A. fumigatus*, to where the entire particle is fully enveloped within the RAW cell, the entire process takes ∼3.5 minutes. A 3D volumetric rendering and rotation of the RAW cell with the internalized *A. fumigatus* confirmed complete uptake (Fig. 5B, Fig. S1, and Video S6).

The optical trap was utilized to hold a pathogen or microbead immobile while the microscope stage was moved to place the object at the desired location. The speed at which the stage moved depended on the type of the object trapped by the optical tweezer. The stage's speed was adjusted such that the drag force created by the fluid flow around the trapped object did not exceed the maximum trapping force. Several characteristics of an object can influence the strength of an optical trap upon it, including size, shape, and the refractive index of the trapped material compared to its surrounding environment. In this report, we trapped both 3 µm (*A. fumigatus*) and 5 µm objects (*C. albicans*, beads) to test the versatility of this technique to trap and manipulate pathogens of interest.

Movies of three separate trapped particles (polystyrene beads, *C. albicans*, and *A. fumigatus*) were examined to ascertain the *average* velocity that is used to move an object on a microscope slide. The average velocity was estimated by analyzing the time stamps of each frame acquired in a movie sequence (see Materials and Methods section). By calculating the average velocity, the average drag force, and thereby the average force exerted by the optical trap, or the trapping force, can be measured.

The drag force is exerted by the solution in the chamber slide and acts to pull the object out of the optical trap, while the force exerted by the optical trap, the trapping force, works to retain the object. As a particle is trapped, the drag force exerted by the surrounding medium is equal to or less than the trapping force. The force due to viscous drag (F) can be calculated by the Navier-Stokes's equation [38], where a liquid with viscosity η flows past a sphere of a known radius r with velocity v:
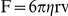



The average drag force was calculated using the average velocity, as described above, and the average trapping force of the optical trap on each particular object was calculated. The trapping force on each object is greater than or equal to the drag force exerted by the surrounding solution. We summarize the average velocity of stage moved around each object and the drag force for each particle (Table 1).

**Table 1:** Average velocity of stage moved around trapped particles.

Trapped particle	velocity (µm/sec)	drag force (pN)	size (µm)
*Candida albicans*	0.29	0.0189	5 µm in length/elliptical
*Aspergillus fumigatus*	0.49	0.0192	3 µm/spherical
Polystyrene beads	0.76	0.0495	5 µm/spherical

The stage had to be moved slower around *C. albicans* compared to a polystyrene bead and *A. fumigatus*, 0.29 µm/sec, in order to keep *C. albicans* in the trap. The calculation for the average speed included both trapped *single C. albicans* pathogens and *dimeric C. albicans* pathogens. To move a dimeric *C. albicans* organism requires slower speeds in order to account for the rotation of the bud around the trapped portion of the particle. The decrease in speed was also due to the forces produced from an elliptical object rather than a true spherical object like the polymeric bead. The speed used to position the stage around *A. fumigatus* was roughly half of what was used to position around polystyrene beads. Although *A. fumigatus* organisms more closely resemble the shape of polystyrene beads compared to *C. albicans* particles, the stage moved at a slower speed due to surface interactions between the pathogen and the coverglass. Despite the difference in speeds, the speeds achieved with pathogenic fungi were more than adequate to move particles to a desired location near antigen presenting cells with ease.

The low laser power used in these experiments to trap an object assured that there was minimal radiation damage to the trapped object and adjacent cell. To increase the trapping force applied to the trapped object, higher laser power can be used. The forces we applied were sufficient to position a fungal pathogen next to a phagocytic cell.

### Real time imaging of synapse formation of Jurkats with CD3 bead

**Figure 6:**
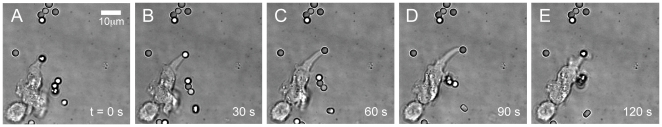
Synapse formation of Jurkat cell to trapped polystyrene bead coated with anti-CD3. (a–e) Brightfield images of Jurkat cell shows that the cell has already formed a synapse with the trapped anti-CD3 bead at 0 s. The Jurkat's protrusion actually pushes the bead out of the trap (at 30 s to 90 s) and starts to bring the bead back to the cell (at 120 s). Movie of synapse formation can be seen in Video S7.

To apply our trapping-spinning disk confocal method to investigate other intercellular interactions in immunology, we also studied the synapse formation of Jurkat cells with polystyrene beads coated with anti-CD3 antibody. The trapped bead is placed in close proximity to an activated Jurkat cell, and a cellular protrusion has formed towards the anti-CD3 bead. Within ∼2.5 minutes, the protrusion has attached to the microbead, capturing it, and is pulling it towards the cell (Fig. 6 and Video S7), and out of the trap. The bead is pushed and pulled according to the movements of the protrusion, and exhibits behavior similar to primary T cells [39]. Unlike the previous experiment, the trap was left on in order to facilitate capture of the bead by the Jurkat's protrusion.

## Discussion

To understand the early steps in intercellular interactions, such as phagocytosis and synapse formation, it is necessary to observe these cellular processes *in vivo* and monitor the fate of multiple components of these systems. The lack of tools in cellular immunology to conveniently manipulate spatio-temporal interactions in a non-invasive manner has made such studies difficult to conduct. Here we present a combined optical trapping platform integrated with a spinning disk confocal microscope, which allows the ability to control cells in any desired geometry to a specific position while observing multiple imaging channels. We expect that this capability will illuminate the early events during physiologically relevant cell-cell contact including phagocytosis or synapse formation.

Although the pathogens of interest in this article generally had dimensions between 3 µm – 5 µm, optical traps have been used to capture smaller particles in the nanometer scale [40], [41] and live cells as large as 10 µm [42]. Although pathogens of interest to our lab typically have dimensions of 3 µm – 5 µm, the optical tweezer system described here is flexible to trap a large range of sizes. Additionally, not only was this optical trapping system able to capture spherical particles, it was capable of trapping more elliptical particles such as *C. albicans*.

The relationship between the refractive indices of the trapped particle and the surrounding medium is crucial in optical trapping. The gradient force created by an optical trap is based on radiation pressure, which is obtained only for a transparent particle where the index of refraction of the trapped particle is less than that of the surrounding medium. If the situation is reversed, photons would primarily *reflect*, instead of *refract*, through the particle, and the particle would exhibit a repulsive force. Consequently, the particle would be repelled from the laser beam. We see a similar situation when we tried to trap highly pigmented particles such as the common laboratory strain of *Aspergillus fumigatus* Af293.

By eye, *A. fumigatus* Af293 exhibits a dark pigmented color on day 1 of culture, and as the colonies grow, the amount of pigment increases in the conidia. The optical trap showed no effect on these particles, so we hypothesized that the pigment in the organisms affected light refraction through the particle. An albino strain of *A. fumigatus* AF293 was obtained[43] (see Methods section), and, unlike their wildtype counterpart, these particles could be easily trapped using our trapping instrumentation. The difference in optical properties and morphology, as seen in electron micrograph images [43], results in the ability to create a stable trap with the albino strain. This difference in surface structure could also affect the adherence between the pathogen and the surface of the coverglass.

With our method, we investigated two unique intercellular events, phagocytosis and synapse formation, in real-time, and analyzed the time frame and structural changes on the cellular surface. By positioning particles next to a cell, we are able to control and manipulate live pathogens to activate the phagocytic machinery to study the immune response in living cells and monitor the absolute beginning of an event. As a result, the entire time course of a phagocytic event can be accurately measured. We followed phagocytosis of *A. fumigatus* as the pathogen is placed next to a macrophage, and watched the initial membrane changes as the particle was engulfed. We were able to accurately measure the entire time frame of this process by controlling when we wanted the process to start with the placement of the pathogen.

We also extended this technique to observing synapse formation by Jurkat cells. By using the optical trap, synapse formation in real time was observed. Although previous work has used optical trapping to study intercellular immune synapses [34], [42], these studies did not observe the dynamic membrane changes the cell undergoes as it binds to another cell or bead.

In summary, we have combined optical trapping with spinning disk confocal microscopy to acquire dynamic, real-time images to investigate early events in intercellular events, specifically phagocytosis and synapse formation. To our knowledge, this method is the first to control and manipulate a pathogen next to a phagocytic cell, and in conjuction with confocal microscopy, was used to record and visualize the absolute beginning and end of phagocytosis in three dimensions with diffraction-limited spatio-temporal resolution. The method was also utilized to study the real-time formation of cellular synapses engaged with polystyrene beads conjugated with anti-CD3 antibody. Our system provides five excitation wavelengths, including UV excitation, enabling imaging of a variety of fluorophores. The trapping apparatus occupies a 4.5 ft^2^ footprint, able to be integrated on many conventional epifluorescent and confocal microscopes. Future modifications would include additional motors on lenses controlling the optical trap to enable additional flexibility of moving the optical trap in addition to the sample stage as well as moving the trap axially during z-stack acquisition.

## Materials and Methods

### Trapping apparatus

As shown in Fig. 1, the instrument was integrated onto an inverted microscope (Nikon, model Ti-E, Nikon Instruments, Melville, NY) and used one laser for trapping (ChromaLase, 1064 nm, 350 mW, Model #: CLAS-106-STF02-02, Blue Sky Research, Milpitas, CA) and one for fluorescence excitation (Innova 70C Spectrum, Coherent, Santa Clara, CA). Components were built on two breadboards (MB1224 and MB1218, Thorlabs, Newton, NJ) elevated above the optical air table (Technical Manufacturing Corporation, Peabody, MA) in order to steer the trapping beam to the microscope. An electronic shutter with pedal control (Uniblitz, Vincent Associates, Rochester, NY) controls the light to the optical trap. The beam is steered towards the back of the microscope using a singlemode optical fiber (Part #: PMJ-3S3S-1064-6, Oz Optics, Ottawa, Ontario, Canada) mounted onto a fiber positioner (Part #: PAF-X-5-C, Thorlabs). The optical fiber was terminated into a collimator (Part #: HPUCO-23-1064-P-25AC, Oz Optics), which delivered a collimated beam into a handmade telescope consisting of a two lenses each with focal lengths of 150 mm (Part #: AC254-150-B, Thorlabs). The lenses in the telescope expanded the incoming trapping beam to fill the back aperture of the trapping objective lens. The fiber positioner, fiber collimator, and lenses in the telescope were mounted to translation stages capable of X, Y, and Z movements (Part #: M-461-XYZ, Newport Corp., Irvine, CA) in order to accurately position the beam into the back port of the microscope. Upon exiting the telescope, the beam entered the back port of the microscope into the microscope's filter turret, which contained an IR dichroic mirror (Part #: ET750sp-2p8, Chroma, Rockingham, VT) that reflected the laser beam into the back aperture of the objective (100 X, 1.49 NA, oil Immersion) (Nikon). In order to create the trap and produce high quality fluorescence images, a high magnification, high numerical aperture (NA) objective, used for Total Internal Reflection Imaging (TIRF), was used (100X, 1.49 NA, oil immersion, Nikon).

### Imaging: microscope and confocal head

Imaging was performed on a Nikon Ti-E inverted microscope, and a Coherent, 4 W, continuous-wave (CW) laser served as a fluorescence excitation light source. This laser is capable of producing several excitation wavelengths used in standard fluorescence imaging: 488 nm, 514 nm, 568 nm, and 647 nm. Laser output is coupled to an optical fiber, which facilitates the alignment of the laser to the confocal head (CSU-X1, Yokogawa, Sugarland, TX). The confocal head contains two spinning disks: one Nipkow spinning disk containing ∼20,000 pinholes and one containing the same number of microlenses that focuses the excitation laser light to its corresponding pinhole. The configuration inside the confocal head provides scan speeds up to 2,000 frames per second.

Excitation light exits the confocal head and into a side port of the microscope where it is then reflected upwards using a slivered prism through filter tourrets and into the sample. The aforementioned Chroma IR filter dichroic, which sits in the upper filter tourret, allows the transmission of all the excitation and emission light used in standard fluorescence imaging. Note, for the current Nikon Ti-E microscope system, it was required to use the “stage-up” system in order to direct the IR trapping beam into the microscope.

A halogen light source and an air condenser (0.52 NA, Nikon) is used for bright field illumination and the 30 mm working distance permits ease of sample loading onto the stage. The microscope is also equipped with a polarizer (Part #: MEN51941, Nikon) and Wollaston prisms (Part #: MBH76190, Nikon) to acquire differential imaging contrast (DIC) images.

Images are acquired using either one of two available CCD cameras (Hamamatsu, Bridgewater, NJ) attached to the trapping-confocal microscope system: (1) ORCA-ER CCD camera (Model #: C4742-80-12AG), capable of acquiring high resolution images (over 1 million pixels) and (2) EM-CCD (Model #: C9100-13) able to acquire images under low light levels with high quantum efficiency. Emission light from the sample passes through the appropriate emission filters (Semrock, Rochester, NY) in either the Ludl filter wheel (Part #: 99A353, Hawthorne, NY) attached to the ORCA-ER CCD camera or the Sutter filter wheel (Model: LB10-NWE, Novato, CA) attached to the EM-CCD camera. The unwanted IR trapping light was blocked from the camera due to the transmission properties of the aforementioned IR dichroic in addition to the emission filters in front of each CCD camera. Each emission filter was optimized to select specific fluorescence bands and to block IR light that could come from the IR trapping laser.

Imaging was performed on cells plated into chambered coverglass (Lab-Tek/Nunc, ThermoScientific, Rochester, NY), which fit into a custom machined stage insert on the microscope's piezo stage (Prior Instruments, Rockland, MA). The piezo stage is capable of X, Y, and Z movement, the acquisition of Z-stacks, and is controlled by a joystick mechanism.

In order to ensure cell viability over long periods of time for adequate live cell imaging, a temperature-regulated environmental chamber was custom-built by In Vivo Scientific (St. Louis, MO). The environmental chamber kept the imaging environment, which includes the piezo stage, objectives, and filter tourrets, at a constant temperature of 37°C.

The environmental chamber also contains a clear, Plexiglass mini-chamber that fits over the sample that can regulate the amount of CO_2_. A humidified air mixture consisting of 5% CO_2_ is created by mixing 100% CO_2_ in a pressurized cylinder with house air in a mixing chamber, which is then fed through a hose into the Plexiglass mini-chamber. The system's digital controller can adjust the amount of CO_2_ introduced to the sample.

### Cell culture and labeling

#### J774 and RAW 264.7 cell culture

Murine J774 and RAW 264.7 macrophage cell lines were cultured in Dulbecco's modified Eagle's medium (DMEM) containing 10% fetal bovine serum (FBS) (HyClone, ThermoScientific, Logan, UT), 1% penicillin/streptomycin (Gibco, Invitrogen, Carlsbad, CA), and 1% L-glutamine (Gibco, Invitrogen). Cells were grown at 37°C with 5% CO_2_ for two days to obtain log-phase growth of the cells in order to optimize their ability to engulf particles. Two hours prior to imaging, RAW cells were re-plated into the chambered coverglass with DMEM medium containing 20% FBS, 2% penicillin/streptomycin, and 2% L-glutamine.

#### Jurkat cell culture

Jurkat cells were maintained at 37°C and 5% CO2 in Iscove's Modified Dulbecco's Medium (IMDM) supplemented with 10% fetal calf serum, 5×10^−4^ M beta-mercaptoethanol, and 15 µg/ml gentamycin sulfate.

#### *C. albicans* and *A. fumigatus* cell culture

Yeast and fungal pathogens were cultured and grown prior to labeling with fluorescent dye. *C. albicans* (SC5314, gift from Gerald Fink, Whitehead Institute) was grown in YPD (Yeast-Peptone Dextrose) liquid culture containing 100 µg/mL ampicillin overnight in a shaker incubator at 37°C. A conidial, albino phenotype of *A. fumigatus* (B-5233/RGD12-8) was grown on a semi-solid agar media containing SBD (Sabouraud dextrose) at 30°C for 3 days (gift from K. J. Kwon-Chung, NIH).

#### Fluorescent labeling of *C. albicans* and *A. fumigatus*

After cultured, both pathogens were washed in phosphate buffered saline (PBS) 3x at 4000 g for 1 minute. Alexa Fluor 488 (AF488), Alexa Fluor 568 (AF568), and Alexa Fluor 647 (AF647) (Invitrogen) (100 mg/mL in dimethylformamide (DMF)) were separately used to label either *C. albicans* or *A. fumigatus* B-5233/RGD12-8. After 3 PBS washes, the pathogens were suspended in 500 µL of PBS, and 3 µg of dye was mixed with the pathogen at 37°C for 30 minutes. During the labeling/incubation, the pathogens were shaken for 10 sec every 10 min to avoid the pathogens settling in the bottom of the tube. The dye-pathogen mixture was then washed again 3 times in PBS (4000 g for 1 minute) and then kept on ice until imaging experiments.

### Lentiviral transduction

293T cells were cultured in DMEM containing 10% FBS, 1% L-glutamine, and 1% penicillin/streptomycin as described previously[32] and used to generate lentivirus encoding YFP-actin.

In order to transduce J774 cells to exhibit YFP (yellow fluorescent protein)-containing actin, concentrated lentivirus was added to J774 cells, and incubated for 24–48 hours. Efficiency of transduction was monitored by fluorescence of cells as seen through epi-fluorescence microscopy.

### Visualizing uptake by raw cells

In order to adequately visualize the uptake and changes to the plasma membrane by *A. fumigatus*, RAW cells were stained with FM 4–64 (Invitrogen), a lipophilic, membrane staining dye. To prepare FM 4–64 for cell labeling, the dye was dissolved at 5 µg/mL in PBS. To image phagocytosis in live cells, in real-time, media was aspirated from RAW cells that were plated in chambered coverglass, and 300 µL FM 4–64 and *A. fumigatus* were added to the chambered coverglass. After the fungi was pipetted into the sample chamber, the particles were trapped before they bound non-specifically to the coverglass and required additional trapping forces required to move the organism. Consequently, during imaging, there could be extraneous particles floating around the RAW cells.

### CD3 bead conjugation

3 µm goat anti-mouse IgG(Fc) particles (Spherotech, Lake Forest, IL) were coated with mouse anti-Human CD3 (invitrogen) by passive adsorption in accordance with the manufacturer's protocol. 200 µL of 0.5% w/v particles were incubated with 30 µg of anti-CD3 antibody in PBS for 2 hours, with rotation at room temp. The beads were washed 3x in PBS and resuspended at 10% w/v in PBS 0.02% sodium azide and stored at 4°C.

### Timecourse measurements of trapped particles

Real-time movies of different trapped pathogens were analyzed to determine the velocity of the stage moved around a trapped particle. Movies consisted of individual TIF files as frames consolidated into a movie or stack file. Each frame was time-stamped indicating the minutes, seconds, and milliseconds. To determine the distance traveled by a particle, each image was calibrated to a known standard. A line tool was then used by our imaging software (Metamorph, Molecular Devices, Downington, PA) to measure the distance. To determine the travel time, the time stamp was taken from the first frame and the last frame (once the particle had move to the desired location), and the time was subtracted to determine the total elapsed time. The velocity was then calculated by dividing the distance traveled by the time elapsed. A similar technique was used to determine the time course for ingestion of *A. fumigatus* by the RAW cells as well as synapse formation.

## Supporting Information

Figure S1 X, Y, Z plane view of fluorescent RAW cell with ingested *A. fumigatus* with expanded view of cell of interest including notation indicating the cross-sections examined in both the XZ and YZ dimensions. The five XZ dimensions are shown above image field, and the five YZ dimensions are shown to the left of the image field.
(TIF)

Video S1 **Movie of a trapped fluorescent polystyrene bead (red) positioned next to a J774 cell expressing YFP-actin.**
(MOV)

Video S2 **Trapped fluorescent** ***C. albicans*** **moved in a field of other** ***C. albicans*** **labeled of one of three colors: AF488 (blue), AF568 (green), or AF647 (red).** The stage is moved around the trapped, blue CA particle.
(MOV)

Video S3 **Trapped** ***C. albicans*** **with attached, budding daughter cell (labeled with AF647, red) in a highly dense field of AF647-labeled and AF488-labeled (green) organisms.** The stage is maneuvered around the red *C. albicans* in order to position the pathogen in another area of the sample dish.
(MOV)

Video S4 **Trapping and positioning of** ***A. fumigatus*** **next to a phagocytic RAW cell.** Brightfield movie of a trapped *A. fumigatus*, moved and positioned next to a RAW cell. The trapped pathogen is slightly out of focus due to the trap pushing the organism slightly above the focal plane. The trap has sufficient precision to select the desired particle even as another microbe moves close to the trapped organism.
(MOV)

Video S5 **Fluorescence imaging of complete phagocytosis of** ***A. fumigatus*** **by RAW cell.** After the trapped pathogen is placed next to a RAW cell, the phagocytosis process is activated, and *A. fumigatus* is subsequently ingested.
(MOV)

Video S6 **3-D rotation of RAW cell with ingested** ***A. fumigatus*** **.** QTVR video, which is an animation based on the user's mouse movements, enables the user to rotate the RAW cell containing the engulfed *A. fumigatus* pathogen.
(MOV)

Video S7 **Synapse formation of Jurkat cell to trapped polystyrene bead coated with anti-CD3 monoclonal antibody.**
(MOV)

## References

[1] Yamada S, Nelson WJ (2007). Synapses: sites of cell recognition, adhesion, and functional specification.. Annu Rev Biochem.

[2] Grakoui A, Bromley SK, Sumen C, Davis MM, Shaw AS (1999). The immunological synapse: a molecular machine controlling T cell activation.. Science.

[3] Monks CR, Freiberg BA, Kupfer H, Sciaky N, Kupfer A (1998). Three-dimensional segregation of supramolecular activation clusters in T cells.. Nature.

[4] Stuart LM, Ezekowitz RA (2008). Phagocytosis and comparative innate immunity: learning on the fly.. Nat Rev Immunol.

[5] Vyas J, Van der Veen A, Ploegh H (2008). The known unknowns of antigen processing and presentation.. Nat Rev Immunol.

[6] Schroeder F, Kinden D (1983). Measurement of phagocytosis using fluorescent latex beads.. J Biochem Bioph Meth.

[7] Desjardins M, Griffiths G (2003). Phagocytosis: latex leads the way.. Curr Opin Cell Biol.

[8] Khademhosseini A, Langer R, Borenstein J, Vacanti JP (2006). Microscale technologies for tissue engineering and biology.. Proc Natl Acad Sci USA.

[9] Voldman J, Gray ML, Schmidt MA (1999). Microfabrication in biology and medicine.. Annu Rev Biomed Eng.

[10] Hultgren A, Tanase M, Chen C, Meyer G, Reich D (2003). Cell manipulation using magnetic nanowires.. J Appl Phys.

[11] Han S, Nakamura C, Obataya I, Nakamura N, Miyake J (2005). A molecular delivery system by using AFM and nanoneedle.. Biosens Bioelectron.

[12] Ionescu-Zanetti C, Shaw RM, Seo J, Jan Y-N, Jan LY (2005). Mammalian electrophysiology on a microfluidic platform.. Proc Natl Acad Sci USA.

[13] Brau R, Tarsa P, Ferrer J, Lee P, Lang M (2006). Interlaced optical force-fluorescence measurements for single molecule biophysics.. Biophys J.

[14] Ashkin A, Dziedzic J, Bjorkholm J, Chu S (1986). Observation of a single-beam gradient force optical trap for dielectric particles.. Opt Lett.

[15] Ashkin A (1997). Optical trapping and manipulation of neutral particles using lasers.. Proc Natl Acad Sci USA.

[16] Ashkin A (1970). Acceleration and trapping of particles by radiation pressure.. Phys Rev Lett.

[17] Ashkin A, Dziedzic J (1987). Optical trapping and manipulation of viruses and bacteria.. Science.

[18] Wang Y, Botvinick E, Zhao Y, Berns M, Usami S (2005). Visualizing the mechanical activation of Src.. Nature.

[19] Khalil AS, Ferrer JM, Brau RR, Kottmann ST, Noren CJ (2007). Single M13 bacteriophage tethering and stretching.. Proc Natl Acad Sci USA.

[20] Khalil AS, Appleyard DC, Labno AK, Georges A, Karplus M (2008). Kinesin's cover-neck bundle folds forward to generate force.. Proc Natl Acad Sci USA.

[21] Li Z, Anvari B, Takashima M, Brecht P, Torres JH (2002). Membrane tether formation from outer hair cells with optical tweezers.. Biophys J.

[22] Kim S, Takeuchi K, Sun Z, Touma M, Castro C (2009). The αβ T cell receptor is an anisotropic mechanosensor.. J Biol Chem.

[23] Mohanty S, Mohanty K, Gupta P (2005). Dynamics of Interaction of RBC with optical tweezers.. Opt Express.

[24] Grashoff C, Hoffman B, Brenner M, Zhou R (2010). Measuring mechanical tension across vinculin reveals regulation of focal adhesion dynamics.. Nature.

[25] Heinrich M, Tian A, Esposito C, Baumgart T (2010). Dynamic sorting of lipids and proteins in membrane tubes with a moving phase boundary.. Proc Natl Acad Sci USA.

[26] Goksör M, Enger J, Hanstorp D (2004). Optical manipulation in combination with multiphoton microscopy for single-cell studies.. Appl Optics.

[27] Hoffmann A, Meyer zu Hörste G, Pilarczyk G, Monajembashi S, Uhl V (2000). Optical tweezers for confocal microscopy.. Appl Phys B-Lasers O.

[28] Lin SJ, Schranz J, Teutsch SM (2001). Aspergillosis case-fatality rate: systematic review of the literature.. Clin Infect Dis.

[29] Wey SB, Mori M, Pfaller MA, Woolson RF, Wenzel RP (1988). Hospital-acquired candidemia. The attributable mortality and excess length of stay.. Arch Intern Med.

[30] Kress H, Stelzer EHK, Griffiths G, Rohrbach A (2005). Control of relative radiation pressure in optical traps: application to phagocytic membrane binding studies.. Phys Rev E Stat Nonlin Soft Matter Phys.

[31] Kress H, Stelzer EHK, Holzer D, Buss F, Griffiths G (2007). Filopodia act as phagocytic tentacles and pull with discrete steps and a load-dependent velocity.. Proc Natl Acad Sci USA.

[32] Artavanis-Tsakonas K, Love JC, Ploegh HL, Vyas JM (2006). Recruitment of CD63 to Cryptococcus neoformans phagosomes requires acidification.. Proc Natl Acad Sci USA.

[33] Blander JM (2008). Phagocytosis and antigen presentation: a partnership initiated by Toll-like receptors.. Ann Rheum Dis.

[34] Oddos S, Dunsby C, Purbhoo MA, Chauveau A, Owen DM (2008). High-speed high-resolution imaging of intercellular immune synapses using optical tweezers.. Biophys J.

[35] Neuman K, Chadd E, Liou G, Bergman K, Block S (1999). Characterization of photodamage to escherichia coli in optical traps.. Biophys J.

[36] Champion JA, Mitragotri S (2006). Role of target geometry in phagocytosis.. Proc Natl Acad Sci USA.

[37] Gudlaugsson O, Gillespie S, Lee K, Vande Berg J, Hu J (2003). Attributable mortality of nosocomial candidemia, revisited.. Clin Infect Dis.

[38] Batchelor G (2000). An Introduction to Fluid Dynamics..

[39] Qi H, Cannons JL, Klauschen F, Schwartzberg PL, Germain RN (2008). SAP-controlled T-B cell interactions underlie germinal centre formation.. Nature.

[40] Ke P, Gu M (1999). Characterization of trapping force on metallic Mie particles.. Appl Opt.

[41] Svoboda K, Block SM (1994). Optical trapping of metallic Rayleigh particles.. Opt Lett.

[42] Mcnerney GP, Hübner W, Chen BK, Huser T (2010). Manipulating CD4+ T cells by optical tweezers for the initiation of cell-cell transfer of HIV-1.. J Biophoton.

[43] Tsai HF, Chang YC, Washburn RG, Wheeler MH, Kwon-Chung KJ (1998). The developmentally regulated alb1 gene of Aspergillus fumigatus: its role in modulation of conidial morphology and virulence.. J Bacteriol.

