# Supplementary figures and images for: Control and Manipulation of Pathogens with an Optical Trap for Live Cell Imaging of Intercellular Interactions
Source: PLoS One. 2010 Dec 31;5(12):e15215. doi: 10.1371/journal.pone.0015215 (PMC3013098; doi:10.1371/journal.pone.0015215)

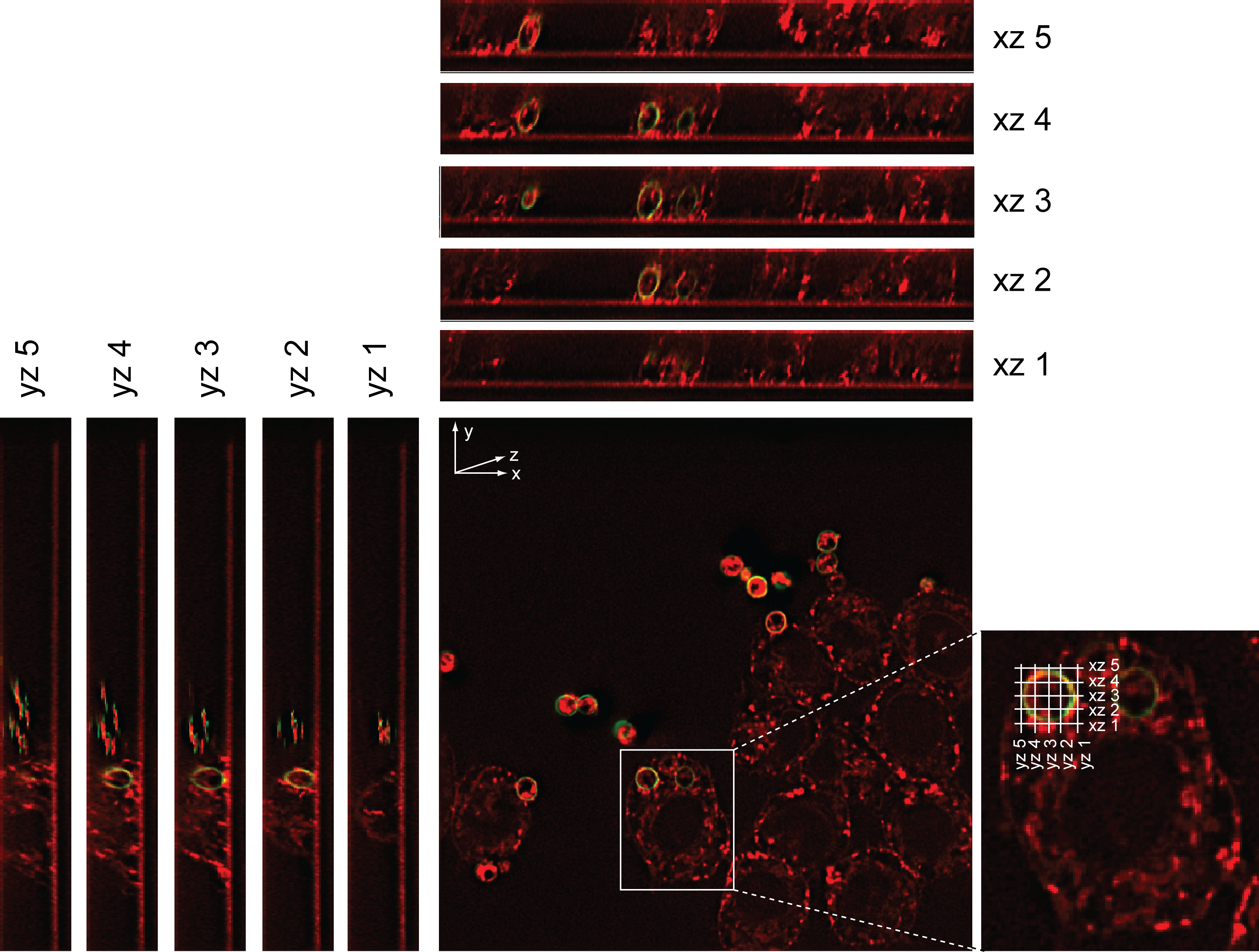

Supplement: Figure S1 — X, Y, Z plane view of fluorescent RAW cell with ingested A. fumigatus with expanded view of cell of interest including notation indicating the cross-sections examined in both the XZ and YZ dimensions. The five XZ dimensions are shown above image field, and the five YZ dimensions are shown to the left of the image field. (TIF) [file pone.0015215.s001.tif]
